# Tandem Use of OvMANE1 and Ov-16 ELISA Tests Increases the Sensitivity for the Diagnosis of Human Onchocerciasis

**DOI:** 10.3390/life11121284

**Published:** 2021-11-23

**Authors:** Cabirou Mounchili Shintouo, Stephen Mbigha Ghogomu, Robert Adamu Shey, An Hotterbeekx, Emel Yagmur, Tony Mets, Luc Vanhamme, Robert Colebunders, Jacob Souopgui, Rose Njemini

**Affiliations:** 1 Department of Gerontology, Faculty of Medicine and Pharmacy, Vrije Universiteit Brussel, Laarbeeklaan 103, 1090 Brussels, Belgium; Cabirou.Mounchili.Shintouo@vub.be; 2 Frailty in Ageing Research Group, Vrije Universiteit Brussel, Laarbeeklaan 103, 1090 Brussels, Belgium; 3 Department of Biochemistry and Molecular Biology, Faculty of Science, University of Buea, Buea P.O Box 63, Cameroon; stephen.ghogomu@ubuea.cm (S.M.G.); sheynce@gmail.com (R.A.S.); 4 Global Health Institute, University of Antwerp, 2610 Antwerp, Belgium; an.hotterbeekx@uantwerpen.be (A.H.); robert.colebunders@uantwerpen.be (R.C.); 5 Department of Molecular Biology, Institute of Biology and Molecular Medicine, IBMM, Gosselies Campus, Université Libre de Bruxelles, 126040 Gosselies, Belgium; emel.yagmur@ulb.be (E.Y.); Luc.Vanhamme@ulb.be (L.V.); 6 Department of Geriatric Medicine, Universitair Ziekenhuis Brussel, Laarbeeklaan 101, 1090 Brussels, Belgium; Tony.Mets@vub.be

**Keywords:** *Onchocerca volvulus*, OvMANE1, Ov-16, ELISA, diagnostic, sensitivity, antibodies

## Abstract

The current serological test for human onchocerciasis relies on IgG4 reactivity against the parasite Ov-16 antigen, with reported sensitivities of only 60–80%. As control programs move from control to elimination, it is imperative to identify novel molecules that could improve the serodiagnosis reliability of this disease. In this study we compared the sensitivity of total IgG against OvMANE1—a chimeric antigen previously identified as a potential biomarker of human onchocerciasis—with that of an Ov-16 antibody test to detect an *Onchocerca volvulus* infection in persons presenting with microfilaria in skin snips. One hundred and ninety serum samples were obtained from persons with epilepsy in an onchocerciasis-endemic area at Ituri in the Democratic Republic of Congo where ivermectin has never been distributed. Fifty-nine (31.1%) samples were from individuals with a positive skin snip test; 41 (69.5%) of these 59 samples were positive with the OvMANE1 test and 41 (69.5%) with the Ov-16 test; 30 (50.8%) samples were positive for both tests and in 52 (88.1%) at least one of the tests was positive. Testing the 131 sera from persons with a negative skin snip result revealed that 63 (48.1%) were positive exclusively with the OvMANE1 test, 13 (9.9%) exclusively with the Ov-16 test and 25 (19.1%) with both tests. Nine European samples from individuals without past travel history in onchocerciasis endemic zones and 15 samples from Rwanda, a hypoendemic country for onchocerciasis were all negative for the OvMANE1 and Ov-16 tests. However, the specificity of both tests was difficult to determine due to the lack of a gold standard for antibody tests. In conclusion, the tandem use of OvMANE1 and Ov-16 tests improves the sensitivity of detecting *Onchocerca volvulus* seropositive individuals but, the OvMANE1 test needs to be further evaluated on samples from a population infected with other helminths to cautiously address its specificity.

## 1. Introduction

Among the 20 Neglected Tropical Diseases recognized by the World Health Organization (WHO, Geneva, Switzerland) as diseases of global public health importance is onchocerciasis—a vector-borne parasitic disease caused by infection with the filarial nematode *Onchocerca volvulus* (Ov; *O. volvulus*) [1,2]. About 21 million people are reported to be infected with *O. volvulus* among which 1.15 and 14.6 million have vision loss and skin disease respectively [3]. Moreover, onchocerciasis may also induce epilepsy [4,5,6]. Furthermore, close to 217.2 million people living in 30 countries mostly in sub-Saharan Africa are in need of preventive chemotherapy for the disease [7].

Currently, the standard method for the diagnosis of active *O. volvulus* infection is the skin snip test. This test demonstrates the presence of *O. volvulus* microfilariae in skin snips by microscopy, a practice that is now widely frowned upon because it is invasive and unable to detect mild infection, besides the challenges to make the difference between *O. volvulus* and other filarial microfilariae [8]. Even though the sensitivity of the test can be increased through amplification of DNA that is extracted from skin snips by polymerase chain reaction (PCR), this method is not field-applicable [9]. Furthermore, the test cannot diagnose onchocerciasis patients during the pre-patent period—the interval between the onset of infection and the development of microfilaria in the skin—which is thought to be between 9 and 15 months [10]. Accordingly, a lot of false negatives are foreseen even in endemic areas where ivermectin treatment has been going on. Therefore, the skin snip test is not considered to have any role in the assessment of onchocerciasis elimination [11], which is currently the public health goal of onchocerciasis programs [12].

The only test approved by WHO that uses human samples for the evaluation of onchocerciasis elimination is the Ov-16 antibody test [13]. This immunological test has a high specificity (>99%) but only a moderate sensitivity (60–80%) [14,15]. It has also been reported that about 15–25% of persons have a genetic limitation that prevents them from generating an immune response to the Ov-16 antigen [16]. Hence, approximately 20% of infected persons will be diagnosed as negative using the Ov-16 test, due to genetic restriction. Therefore, for a complete assessment of onchocerciasis elimination, more sensitive tests which can be employed singly or in combination with the Ov-16 test must be developed [17].

To identify alternative biomarkers, the OvMANE1 chimeric antigen was engineered and validated as a sensitive and specific biomarker for onchocerciasis diagnosis, using immune-dominant peptides of the parasite [18]. Moreover, the possibility of cross-reaction with OvMANE1 chimeric antigen in individuals infected with other related nematodes was investigated—using serum samples from patients infected with *Brugia malayi, Mansonella perstans, Ascaris lumbricoides* or *Wuchereria bancrofti*—and OvMANE1 chimeric antigen significantly discriminated onchocerciasis sera from that of related nematodes [18], suggesting OvMANE1 chimeric antigen as a potential biomarker for onchocerciasis diagnosis. In this study, we compared the sensitivity of total IgG against the OvMANE1 test with that of an Ov-16 antibody test, using serum samples obtained from persons with epilepsy in an onchocerciasis endemic area in the Democratic Republic of Congo where ivermectin was never distributed. Our investigations led to the suggestion that tandem use of OvMANE1 and Ov-16 tests improves the sensitivity of detecting *O. volvulus* seropositive individuals.

## 2. Materials and Methods

### 2.1. Ethical Considerations

Approval for this study was obtained from the ethics committees of the University of Kinshasa’s School of Public Health, Democratic Republic of Congo (permission number: ESP/CE/013/2018), the University of Antwerp, Belgium (approval number: B300201733350) and the Cameroon Bioethics Initiative (CAMBIN) Ethics Review and Consultancy Committee (ERCC) (reference number: CBI/443/ERCC/CAMBIN). All participants voluntarily signed an informed consent form, and only consenting individuals were enrolled. Minors >12 years and <18 years signed an assent form in addition, while parents or legal guardians consented for younger participants. Participants’ identities and other information were kept private.

### 2.2. Study Design and Site

The study was performed in an onchocerciasis-endemic area in the Logo health zone, in the Ituri province, Democratic Republic of Congo, where ivermectin had never been distributed. Samples were obtained during a screening survey of persons with epilepsy for a clinical trial to evaluate the effect of ivermectin on the frequency of seizures in persons with an *O. volvulus* infection [19]. Clinical examination to assess *O. volvulus* infection was conducted for all the participants in this study and the data for all the onchocerciasis patients were published [20]. Control samples were obtained from individuals living in an onchocerciasis hypo-endemic region (HES, n = 15) in Huye, Rwanda [7] and from European individuals (ECS, n = 9). The European population was made up of individuals who have never visited Africa, Yemen or Latin America—where onchocerciasis is endemic—and thus have not been exposed to the parasite [7].

### 2.3. Identification of O. volvulus in Skin Snips by Microscopy

Two skin snips were obtained from the left and right iliac crests of each participant and transferred to separate wells of a microtiter plate using a sterile Holtz corneo-scleral punch (2 mm). A couple of drops of saline was added into each well and incubated for 24 h to allow microfilariae emerge from the snips. Microfilariae were counted under a microscope and the microfilaria load was recorded as number of microfilaria/skin snip.

### 2.4. Diagnosis of O. volvulus Infection by OvMANE1 and Ov-16 ELISA Tests

The gene coding for OvMANE1 chimeric antigen was expressed as an MBP fusion protein (OvMANE1_MBP, 60.4 kDa) in the pMAL-c5X expression vector. Interestingly, the MBP tag was found not to interfere with the immune response of the fusion protein [18]. All sera were screened for OvMANE1 total IgG antibodies by indirect ELISA. Briefly, MaxiSorp 96 well microtiter plates (Nunc, Roskilde, Denmark) were coated overnight at 4 °C with 200 µL OvMANE1_MBP chimeric antigen diluted in PBS at a final concentration of 2 µg/mL. Plates were washed 3 times with wash buffer (PBS + 0.05% Tween 20; PBST) and blocked with SuperBlock buffer (Thermo Fisher Scientific, Merelbeke, Belgium) for 1 h 30 min at room temperature. After three rounds of washes at 5 min interval each, the microtiter plates were incubated with the various serum samples as the primary antibody at a dilution of 1:2000 for 2 h at room temperature. The plates were washed as described above and incubated with goat anti-human IgG (Fc Specific) peroxidase conjugate (Sigma, St. Louis, MI, USA) as the secondary antibody at a dilution of 1:5000 for 1 h 30 min at room temperature. After a final wash, 3,3′,5,5′-tetramethylbenzidine (TMB, Sigma, St. Louis, MI, USA) was added as a chromogenic substrate for 10 min at room temperature. The reactions were stopped with 3 M hydrochloric acid after which the optical density (OD) was read at 450 nm using the iMark microplate reader (BIORAD, Irvine, CA, USA). All antibody dilutions were done in Superblock buffer (Thermo Fisher Scientific, Merelbeke, Belgium).

The same serum samples were also screened for Ov-16 IgG4 antibodies by ELISA as described previously [17]. Briefly, Maxisorp 96 well microtiter plates (Nunc, Roskilde, Denmark), were coated with 100 µL of 1 µg/mL recombinant Ov-16 (diluted in PBS) and incubated overnight at 4 °C. Thereafter, the plates were washed once with 300 µL PBST and non-specific binding sites blocked by incubation with 300 µL of the SuperBlock buffer (Thermo Fisher Scientific, Merelbeke, Belgium) for 1 h at room temperature. After washing, 100 µL of serum, (diluted 1:200 in SuperBlock buffer), was added into each well and incubated for 1 h at room temperature. The plates were washed as above and incubated for 30 min at room temperature with mouse monoclonal anti-human IgG4 Fc (HRP) antibody (Abcam, Cambridge, UK) diluted 1:10,000 in superblock buffer (Thermo Fisher Scientific, Merelbeke, Belgium). The plates were then washed and 100 µL of TMB substrate solution (Sigma, St. Louis, MI, USA) was added into each well and incubated for 10 min at room temperature. The reactions were stopped with 3 M hydrochloric acid and the absorbance measured at 450 nm.

### 2.5. Statistical Analyses

Data were analyzed using Microsoft Excel 2010. The Shapiro-Wilk test was used to assess the normality of distributions. Scatter plots were generated using GraphPad Prism 7.0 (La Jolla, CA, USA) and the data were expressed as median with interquartile range. Comparison of three groups was done using Kruskal Wallis test followed by Dunn’s test for multiple comparisons. The discriminatory performance of total IgG and IgG4 were assessed using receiver operating curve analyses. The area under the receiver operating curve (AUCs) were evaluated using the trapezoid method. A cutoff value was selected based on the Youden’s index and the sensitivities, specificities with 95% confidence intervals were then calculated for the selected cutoff value. Spearman correlation was performed using SPSS V26 (IBM Corp., Armonk, NY, USA) A *p*-value below 0.05 was considered statistically significant.

## 3. Results

### 3.1. Description of Study Population

Serum samples from 190 participants were randomly selected from 387 serum samples obtained during *O. volvulus* infection screening of persons with epilepsy in Ituri province, Democratic Republic of Congo [19]. The serum samples were blinded and used for serological analysis. After unblinding, 59 (31.1%) samples were from individuals with a positive skin snip test and the remaining 131 samples were from persons with negative skin snip results.

### 3.2. Comparison of OvMANE1 and Ov-16 ELISA Tests

Of the 59 serum samples positive for skin snip, 41 (69.5%) were positive with the OvMANE1 test (OD ≥ 0.323) and 41 (69.5%) with the Ov-16 test (OD ≥ 0.035); 30 (50.8%) sera were positive for both ELISA tests and in 52 (88.1%) at least one of the ELISA tests was positive. Regarding the 131 samples from persons with a negative skin snip result, 63 (48.1%) were positive exclusively with the OvMANE1 test, 13 (9.9%) exclusively with the Ov-16 test and 25 (19.1%) were positive for both ELISA tests. 30 (22.9%) serum samples were negative for both ELISA tests (see Table 1).

### 3.3. Humoral Immune Response to OvMANE1 and Ov-16 Antigens

**Figure 1:**
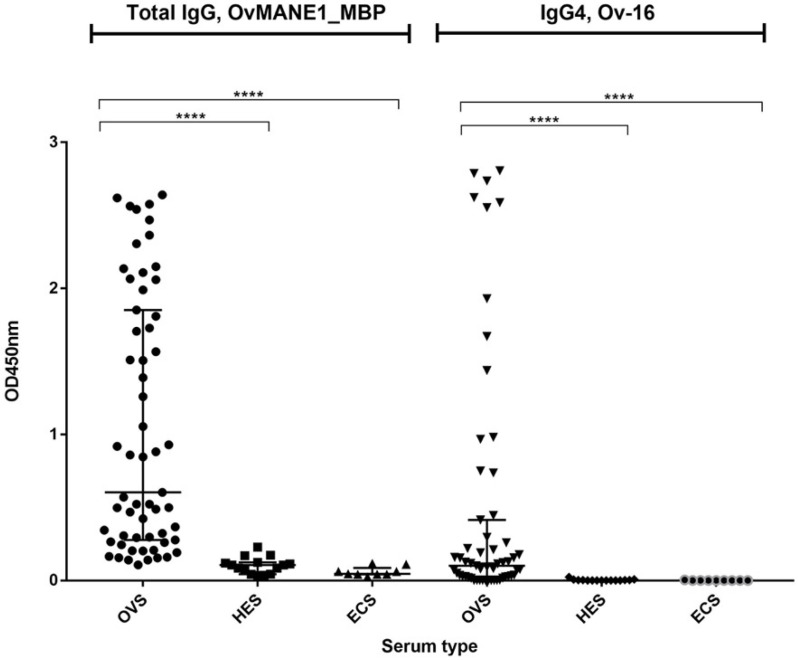
Analysis of humoral immune response to OvMANE1 and Ov-16 antigens using sera from *O. volvulus* infected and non-infected individuals. In separate reactions, OvMANE1_MBP and Ov-16 antigens were used to coat microtiter plates. The plates were blocked and incubated with different serum samples followed by incubation with secondary antibodies, namely goat anti-human IgG (for plates coated with OvMANE1_MBP chimeric antigen) or mouse monoclonal anti-Human IgG4 (for plates coated with Ov-16 antigen), all peroxidase conjugated. The plates were revealed using TMB, the optical density (OD) was read at 450 nm and OD values were plotted against the different serum types. OVS = *O. volvulus* serum (n = 59), HES = Hypo-endemic serum (n = 15), ECS = European control serum (n = 09). The groups were compared using Kruskal Wallis test followed by Dunn’s test for multiple comparisons. **** indicates a significant difference with *p* < 0.0001.

To assess the ability of the OvMANE1 and Ov-16 tests to detect *O. volvulus* seropositive individuals, 59 microfilaria positive samples (OVS) diagnosed by skin snip, 15 Rwandan (HES) and 9 European (ECS) sera were assayed. Both OvMANE1 and Ov-16 tests discriminated between *O. volvulus* infected serum samples and control serum samples from onchocerciasis non-endemic countries (*p* < 0.0001) (see Figure 1). The area under the receiver operating curve (AUC) was 0.9684 for OvMANE1 test and 0.8746 for Ov-16 test. The sensitivity was 71.2 % and 69.5 % for OvMANE1 and Ov-16 tests respectively. The specificity was 100.0% for both tests (see Table 2).

**Figure 2:**
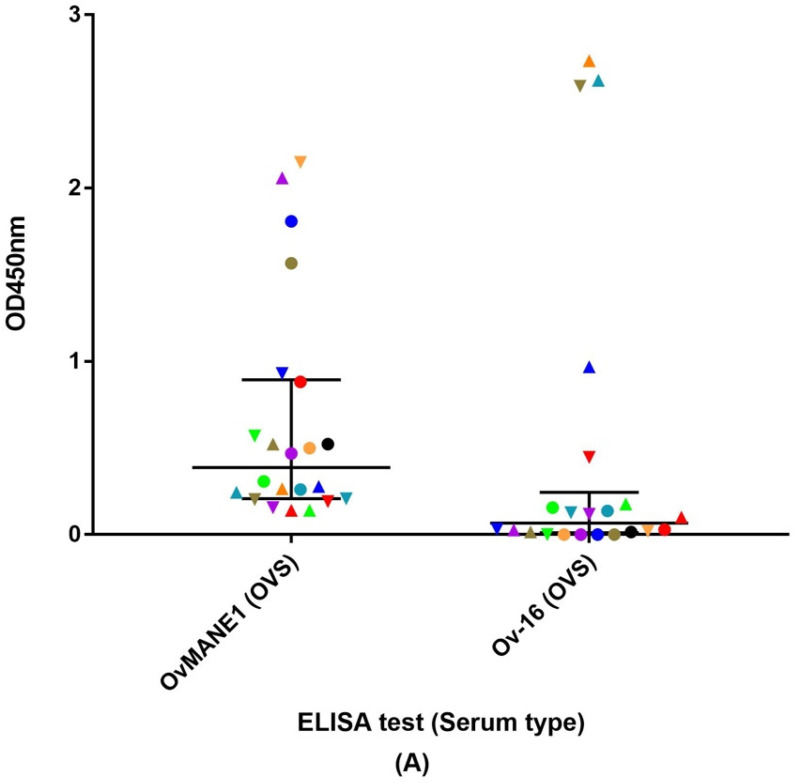

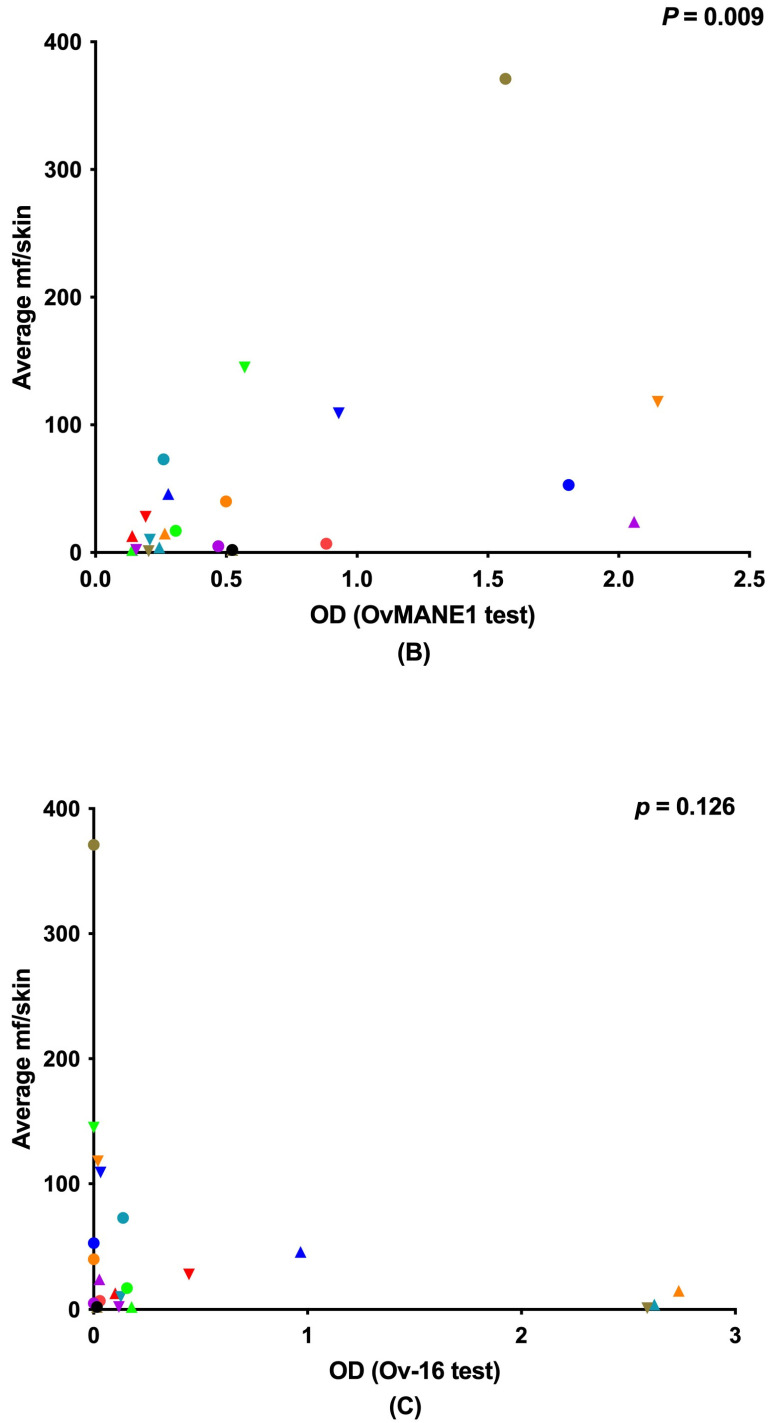
Analysis of humoral immune response to OvMANE1 and Ov-16 antigens using *O. volvulus* serum (OVS, n = 22), which were either positive according to OvMANE1 test (n = 11) but not Ov-16 test (n = 11) and vice versa. (**A**) optical densities (OD) at 450 nm were plotted against OvMANE1 and Ov-16 tests using color codes and shapes to match the specific serum samples in both tests. The average microfilaria (mf) load per skin snip was plotted against (**B**) the OD of OVS in OvMANE1 test and (**C**) the OD of OVS in Ov-16 test using color codes and shapes that matches the specific serum sample. Color codes were assigned to each sample to clearly differentiate the OD of the sample in the different tests.

Furthermore, the 22 serum samples that were positive by skin snip test having amongst them 11 samples which were positive for OvMANE1 test and negative for Ov-16 test and 11 others positive by Ov-16 test but not OvMANE1 test were given a color code and evaluated as indicated in Figure 2. A difference in their OD was revealed between the two tests (Figure 2A). There was a correlation between the average microfilaria load and the OD of the positive samples in the OvMANE1 test (r = 0.542, *p* = 0.009) (see Figure 2B). No correlation was observed between the average microfilaria load and the OD of the positive samples in the Ov16 test (r = −0.336, *p* = 0.126) (see Figure 2C). However, no correlation was observed between the average microfilaria load and the OD of all the samples that were positive by skin snip in the OvMANE1 test (r = 0.153, *p* = 0.248) and Ov-16 test (r = 0.023, *p* = 0.861) (see Figure S1).

A total of 88 from the 190 serum samples were randomly selected and screened for *Strongyloides* by Vieri et al. [19]. All the *Strongyloides* positive samples (n = 10, 11.4%) were positive for onchocerciasis using the skin snip test. Among these positive samples, 7 (70.0%) were positive with the OvMANE1 and Ov-16 tests while 1 (10.0%) was positive for OvMANE1 test alone and 2 (20.0%) for Ov-16 test alone.

## 4. Discussion

Although the entomological evaluation of the presence of *O. volvulus* in black flies (the vector responsible for the transmission of the parasite to humans) and serological assessment of *O. volvulus* infection using the Ov-16 test have been effective for validating suppression and interruption of transmission in the Americas and some foci in Africa, it is difficult to translate the successes of these diagnostic tools to the rest of Africa [21]. The prevalence of onchocerciasis is higher in Africa, which accounts for more than 99% of all infected cases [3]. Hence, the wrong classification of 20–40% of patients infected with *O. volvulus* as negative for onchocerciasis by the Ov-16 test will result to a large population wrongly classified. These persons can serve as active *O. volvulus* parasite reservoirs as opposed to a smaller population of false negative individuals in the Americas with a lower probability to continue transmission. Therefore, novel diagnostic tests are needed that can be used singly or in combination with the Ov-16 test to improve its sensitivity. This would considerably speed up the progress made to eliminate onchocerciasis in Africa.

In this study, the sensitivity of the OvMANE1 and Ov-16 tests was evaluated to determine if the two tests could be used in combination to detect *O. volvulus* seropositive persons presenting with or without microfilaria in their skin snips. Among the 190 serum samples tested for *O. volvulus* infection, 59 (31.1%) samples were from individuals with a positive skin snip test. Forty-one (69.5%) of the 59 samples were positive with the OvMANE1 test and 41 (69.5%) with the Ov-16 test. Thirty (50.8%) of the 59 samples were positive for both tests while 52 (88.1%) were positive for at least one of the tests. A study by Hotterbeekx et al., [17] reported Ov-16 RDT (SD Bioline) results of all the participants in this study among others. Comparison of the Ov-16 RDT (SD Bioline) results with the Ov-16 ELISA results obtained in this study revealed that the specificity and sensitivity of both tests were not identical. This suggests a moderate increase of sensitivity (18.6%) can be obtained by using both tests as compared to the Ov-16 test alone (see Figure 2A). Therefore, the tandem use of OvMANE1 and Ov-16 tests may represent a more appropriate tool for onchocerciasis elimination mapping because of the improved sensitivity. Furthermore, an increased sensitivity may decrease the number of children to be screened to meet the elimination thresholds set by WHO (current sample size is ≥3000) [22]. Correlation analysis between the average microfilaria load and the 22 samples that were either positive for OvMANE1 or Ov-16 tests revealed a correlation between the average microfilaria load and the OD of the positive samples in the OvMANE1 test (r = 0.542, *p* = 0.009) (see Figure 2B). There was no correlation between the average microfilaria load and the OD of the positive samples in the Ov16 test (r = −0.336, *p* = 0.126) (see Figure 2C). However, no correlation was observed between the average microfilaria load and all the *O. volvulus* infected samples by skin snip for OvMANE1 test (r = 0.153, *p* = 0.248) and Ov-16 test (r = 0.023, *p* = 0.861). This non-association of Ov-16 and OvMANE1 antibodies with microfilariae load cannot be explained by ivermectin use as all study participants were ivermectin naïve. An explanation might be due to the matrix (blood or skin snip), as microfilaria are in the skin and antigens might not make it into the bloodstream that easily. Notwithstanding, based on the Ov-16 test, the majority of the samples (134/190; 71%) were correctly classified as positive or negative for microfilaria.

Of the 131 sera from persons with a negative skin snip result, 63 (48.1%) were positive exclusively with the OvMANE1 test, 13 (9.9%) exclusively with the Ov-16 test and 25 (19.1%) with both tests. The specificity of both tests is difficult to determine due to the lack of a gold standard for the antibody test. However, since both tests are antibody based and thus unable to differentiate between past and active infection [23], they may have diagnosed these individuals as positive because the individuals may have been infected with the parasite in the past. False positive results will be obtained from individuals who have been cured but still have antibodies against OvMANE1 and Ov-16 antigens. Hence, the tests will diagnose these individuals to be positive for onchocerciasis because of the presence of these antibodies. Consistently, up to 5% of the inhabitants leaving in endemic areas for onchocerciasis do not develop any sign or symptom [24,25]. Hence, there is also a need for an antigen capture test to be added in the diagnostic toolbox of human onchocerciasis. Furthermore, the decrease in sensitivity of the skin snip test to diagnose *O. volvulus* infection in persons with very low microfilaria load and its inability to diagnose onchocerciasis patients during pre-patent period [10] may also contribute to the discrepancy between result obtained by the skin snip test and the antibody base tests. Additionally, a limitation of this study that may contribute to the differences in result obtained by the skin snip test and the antibody base tests is the fact that the negative samples by skin snips were not diagnosed for the presence of the parasite DNA by PCR. Reports indicate that molecular detection of *O. volvulus* in skin snips has a higher sensitivity compared to the detection of the parasite in skin snips by microscopy [26,27].

Nine European and 15 Rwandan samples from a non-onchocerciasis endemic region were all negative for the OvMANE1 and Ov-16 tests. This implies that both tests could specifically differentiate between onchocerciasis and non-onchocerciasis samples. However, there is a need to further evaluate the specificity of OvMANE1 test in a larger population in communities that are non-endemic for onchocerciasis but endemic for other helminth infections. Nonetheless, upon analysis of OvMANE1 test results with 88 serum samples that were tested for *Strongyloides* by Vieri et al. [19], no evidence of cross reactivity between OvMANE1 chimeric antigen and antibodies with persons infected with *Strongyloides was found*. Furthermore, analysis of serum samples collected in Huye, Rwanda did not reveal a cross reactivity between OvMANE1 chimeric antigen and antibodies from other helminths including *Strongyloides* that has been reported at the time of sample collection to be on an increase trend in the southern part of Rwanda [28].

In conclusion, the tandem use of OvMANE1 and Ov-16 tests improves the sensitivity to detect *O. volvulus* infection. This advocates the use of a multi antigenic diagnostic test in the future and pleads for the characterization of extra *O. volvulus* antigens in the context of diagnosis. However, OvMANE1 test needs to be further evaluated using samples from communities harboring individuals infected with other helminths but not onchocerciasis to further determine cautiously its specificity.

## Figures and Tables

**Table 1:** Comparison of OvMANE1 and Ov-16 tests with skin snip test.

	Skin Snip	OvMANE1 ELISA	Ov-16 ELISA	Total
All positive	+	+	+	30
Ov-16 ELISA negative only	+	+	-	11
OvMANE1 ELISA negative only	+	-	+	11
OvMANE1 ELISA + Ov-16 ELISA both negative	+	-	-	7
OvMANE1 ELISA positive only	-	+	-	63
Ov-16 ELISA positive only	-	-	+	13
Skin snip negative only	-	+	+	25
All negative	-	-	-	30

+: positive test result; -: negative test result

**Table 2:** Receiver operating curve (ROC) values for IgG responses to OvMANE1 and Ov-16 antigens and their diagnostic accuracy parameter.

		Total IgG (OvMANE1)	Total IgG4 (Ov-16)
ROC curve analysis	ROC curve area (AUC)	0.9684	0.8746
	95% CI of AUC	0.9320 to 1.000	0.7967 to 0.9524
	*p*-value	<0.0001	<0.0001
Diagnostic Accuracy Parameter	Cut off value	0.323	0.035
	Sensitivity (%) (95% CI)	71.2 (58.6% to 81.2%)	69.49 (56.9% to 79.8%)
	Specificity (%) (95% CI)	100.0 (79.6% to 100.0%)	100.0 (79.6% to 100.0%)

## Data Availability

The datasets generated during this study are available from the corresponding authors on reasonable request.

## Supplementary Materials

The following are available online at https://www.mdpi.com/article/10.3390/life11121284/s1, Figure S1: Analysis of humoral immune response to OvMANE1 and Ov-16 antigens using *O. volvulus* serum (OVS, n = 59).
