# Supplementary material for: Tandem Use of OvMANE1 and Ov-16 ELISA Tests Increases the Sensitivity for the Diagnosis of Human Onchocerciasis
Source: Life (Basel). 2021 Nov 23;11(12):1284. doi: 10.3390/life11121284 (PMC8703281; doi:10.3390/life11121284)
Supplement: Supplementary file 1 [file life-11-01284-s001.zip › life-1447445-supplementary.pdf]

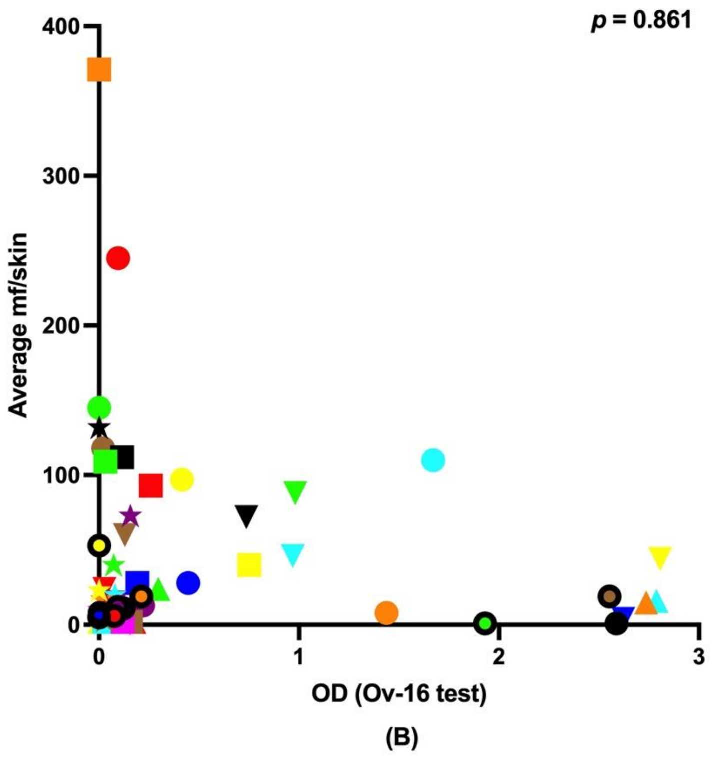

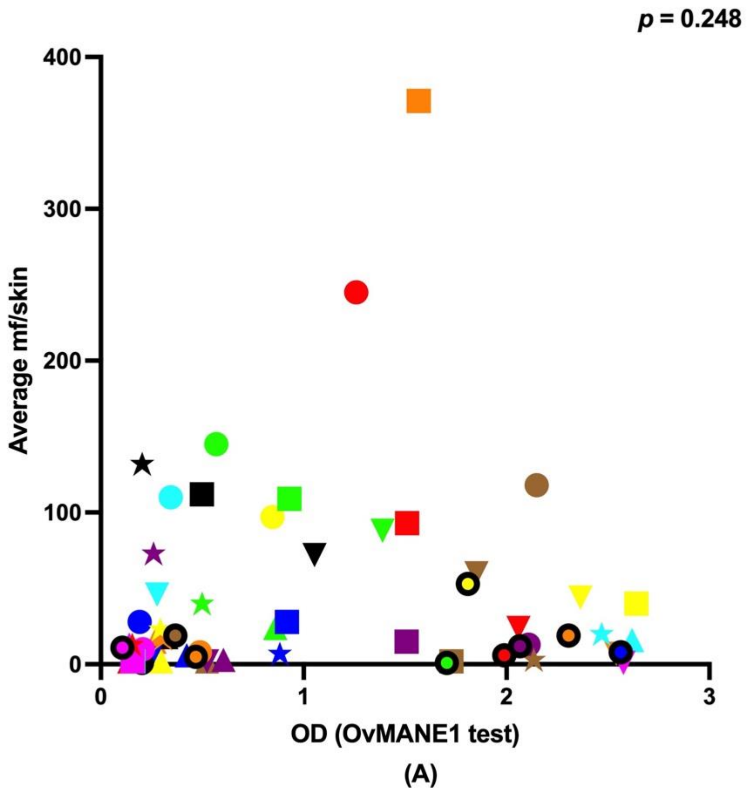

Figure S1. Analysis of humoral immune response to OvMANE1 and Ov-16 antigens using *O. volvulus* serum (OVS, n = 59). The average microfilaria (mf) load per skin snip was plotted against A) the OD of all OVS in OvMANE1 test and B) the OD of all OVS in Ov-16 test using color codes and shapes that matches the specific serum sample. Color codes were assigned to each sample to clearly differentiate the OD of the sample in the different tests.
